# Doxorubicin–Loaded Human Serum Albumin Submicron Particles: Preparation, Characterization and In Vitro Cellular Uptake

**DOI:** 10.3390/pharmaceutics12030224

**Published:** 2020-03-02

**Authors:** Saranya Chaiwaree, Ausanai Prapan, Nittiya Suwannasom, Tomás Laporte, Tanja Neumann, Axel Pruß, Radostina Georgieva, Hans Bäumler

**Affiliations:** 1 Institute of Transfusion Medicine, Charité-Universitätsmedizin Berlin, 10117 Berlin, Germany; mam.chaiwaree@gmail.com (S.C.); ausanaip@nu.ac.th (A.P.); nittiya.su@up.ac.th (N.S.); tomas.laporte93@gmail.com (T.L.); axel.pruss@charite.de (A.P.); radostina.georgieva@charite.de (R.G.); 2 Department of Pharmaceutical Technology, Faculty of Pharmacy, Payap University, Chiang Mai 50000, Thailand; 3 Department of Radiological Technology, Faculty of Allied Health Sciences, Naresuan University, Phitsanulok 65000, Thailand; 4 Division of Biochemistry, School of Medical Sciences, University of Phayao, Phayao 56000, Thailand; 5 Instituto de Nanosistemas, Universidad Nacional de San Martín, San Martín, Pcia de Buenos Aires 1021, Argentina; 6 JPK BioAFM Business, Nano Surfaces Division, Bruker Nano GmbH, 12489 Berlin, Germany; Tanja.Neumann@bruker.com; 7 Department of Medical Physics, Biophysics and Radiology, Medical Faculty, Trakia University, 6000 Stara Zagora, Bulgaria

**Keywords:** doxorubicin, albumin particles, CCD technique, cellular uptake, submicron particles

## Abstract

Doxorubicin (DOX) is an effective anthracycline antibiotic drug which is commonly used in a broad range cancer therapy. However, due to dose depending side effects and toxicity to non-cancerous tissues, its clinical applications are restricted. To overcome these limitations, human serum albumin (HSA) has been investigated as a biocompatible drug delivery vehicle. In this study, human serum albumin submicron particles (HSA-MPs) were fabricated by using the Co-precipitation–Crosslinking–Dissolution technique (CCD technique) and DOX was loaded into the protein particles by absorption. DOX-HSA-MPs showed uniform peanut-like shape, submicron size and negative zeta-potential (−13 mV). The DOX entrapment efficiency was 25% of the initial amount. The in vitro release in phosphate buffered saline pH 7.4 was less than 1% within 5 h. In contrast, up to 40% of the entrapped DOX was released in presence of a protein digesting enzyme mixture (Pronase^®^) within the same time. In addition, in vitro cytotoxicity and cellular uptake of DOX-HSA-MPs were evaluated using the lung carcinoma cell line A549. The results demonstrated that DOX-HSA-MPs reduced the cell metabolic activities after 72 h. Interestingly, DOX-HSA-MPs were taken up by A549 cells up to 98% and localized in the cell lysosomal compartment. This study suggests that DOX-HSA-MPs which was fabricated by CCD technique is seen as a promising biopolymer particle as well as a viable alternative for drug delivery application to use for cancer therapy.

## 1. Introduction

Doxorubicin (DOX) was first extracted from *Streptomyces peucetius var. caesius* in the 1970s [1]. It has been one of the most effective drugs in an anthracycline antibiotic which is used to treat several types of cancer such as lung or breast cancer. Several mechanisms have been proposed to explain the cytotoxic activity of DOX. However, 2 major mechanisms are under discussion: intercalating into DNA, thereby inhibiting the topoisomerase II leading to DNA synthesis inhibition; and generation of free radicals leading to DNA and cell membrane damage [1,2,3]. However, DOX is also known to be cardiotoxic [4] and its clinical application is limited by detrimental effect on normal tissues including brain, kidney, liver [5,6] and skin [7]. Furthermore, as conventional chemotherapeutic drug DOX exhibits high toxicity due to the limited accessibility of the drug by the tumor cell. To avoid these effects, various types of drug carriers have been developed. Currently, pegylated liposomal doxorubicin (Doxil^®^, Lipodox^®^) and unpegylated (Myocet^®^) (FDA-approved) [8], encapsulated dextran–DOX conjugate using chitosan nanoparticles [9], DOX-loaded mesoporous silica nanoparticle [10] and Dox-encapsulated in micelles or nanoemulsion [11] are being investigated as possible candidates. Most of these formulations showed a lower incident of cardiotoxicity and myelosuppression when compared with conventional ones [12].

In recent years, albumin has been actively explored and considered as a reliable and efficient carrier for drug delivery systems [13,14,15]. Additionally, the tumor cells express albumin receptors [16]. Albumin is the most abundant protein in the blood plasma accounting for 60% of the total plasma protein content [17,18]. This protein can bound various hydrophobic and hydrophilic therapeutics forming a stable complex with high drug loading and entrapment efficiency [15,16]. Furthermore, human serum albumin (HSA) is very attractive for application in drug delivery systems due to its high solubility, stability to pH changes (between pH 4 to 9) [19], good biocompatibility, biodegradability, low toxicity and non-immunogenic properties. It is modifying the distribution of drugs in tissues and improving their intracellular distribution [20,21].

In our group a successful technique, known as Co-precipitation–Crosslinking–Dissolution (CCD) technique was developed, which was used to fabricate biopolymer particles [22,23,24]. This approach has been shown to entrap proteins with high effectiveness using appropriate inorganic compounds for the precipitation [25]. More recently, HSA submicron particles produced by this technique have been successfully loaded with riboflavin [26].

In the present study, we propose a procedure to fabricate doxorubicin loaded albumin microparticles (DOX-HSA-MPs) by a slightly modified CCD-Technique, which releases Doxorubicin only after endocytosis and digestion of the particles. The hypothesis was tested with pulmonary adenocarcinoma cells (A459 cell line) presenting albumin receptors and human bronchial epithelial cells (BEAS-2B cell line), without albumin receptors. The obtained DOX-HSA-MPs were characterized concerning size, morphology, surface charge as well as for their drug release profile, cellular uptake by A459 and BEAS-2B cells as well as for cytotoxicity to these cells.

## 2. Materials and Methods

### 2.1. Materials

Doxorubicin hydrochloride (DOX) was purchased from Cayman chemical (Ann Arbor, MI, USA). Human serum albumin solution (200 g/L HSA, 145 mM NaCl) was purchased from Grifols Deutschland GmbH (Frankfurt, Germany). Dimethyl sulfoxide (DMSO), ethylene diamine tetraacetic acid disodium salt (Na_2_·EDTA), glutaraldehyde (GA), magnesium chloride (MnCl_2_), sodium carbonate (Na_2_CO_3_), phosphate buffered saline (PBS), pH 7.4, fluorescein isothiocyanate (FITC), glycine and sodium borohydride (NaBH_4_) were purchased from Sigma-Aldrich (Munich, Germany). Sodium hydroxide (NaOH) was purchased from Carl Roth GmbH (Karlsruhe, Germany). Ampuwa and sterile 0.9% NaCl solution were purchased from Fresenius Kabi Deutsch-land GmbH (Bad Homburg, Germany). Pronase^®^ was purchased from Roche Diagnostics GmbH (Mannheim, Germany). The pulmonary adenocarcinoma cell line A549 was kindly provided by Prof. Sergio Moya, CIC bioma GUNE, San Sebastian, Spain. The human normal bronchial epithelium cell line BEAS-2B was purchased from Sigma-Aldrich Chemie GmbH (Munich, Germany). Fetal bovine serum (FBS) was purchased from Biochrom GmbH (Berlin, Germany), RPMI 1640 was purchased from Corning New York, NY, USA). Cell Counting Kit-8 (CCK-8) was purchased from Dojindo EU GmbH (Munich, Germany). Lysotracker^®^ Deep Red was purchased from Invitrogen (Thermofisher, Waltham, MA, USA). 

### 2.2. Particles Fabrication

**Figure 1:**
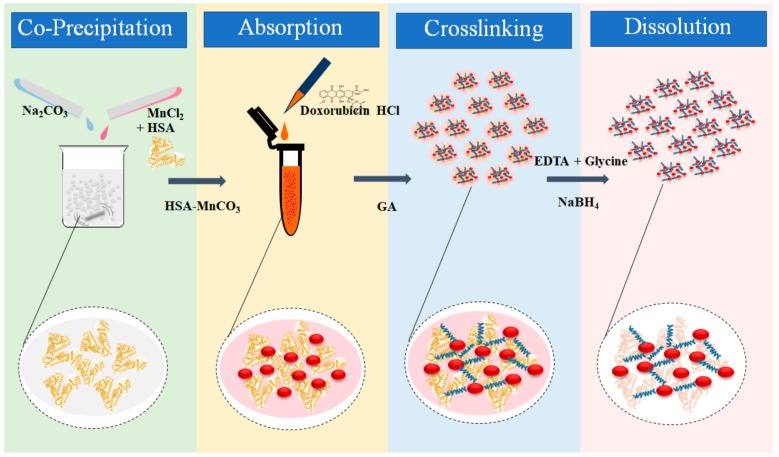
Schematic of the fabrication of the doxorubicin (DOX)-human serum albumin (HSA)-microparticles (MPs) via a modified Co-precipitation–Crosslinking–Dissolution (CCD) technique.

DOX-HSA-MPs, were fabricated by a slightly modified CCD technique as previously described [22,23,24] (Figure 1). Equal volumes of 0.125 M of MnCl_2_ containing 0.5% HSA of 0.125 M of Na_2_CO_3_ were mixed rapidly for 30 s under vigorous stirring using a mechanical stirrer (model IKA RW 20 DZM equipped with a 35-mm-diameter rotor) at room temperature. Then, 0.05% of HSA solution was added to the suspension and incubated for further 5 min under stirring to prevent agglomeration of the particles. The suspension was centrifuged and the particles were washed twice (3000× *g* for 3 min). The resulting particle suspension was adjusted to a volume concentration of 30% and incubated with 10 mg/mL DOX in 50% DMSO for 1 h. After the incubation, the supernatant was collected and the particles were washed with 0.9% NaCl (3000× *g* for 3 min) until the supernatant became colorless. The resulting particles (30% volume concentration) were incubated with 0.1% GA solution for 1 h at ambient temperature allowing the cross-linking reaction to perform in the formulated submicron particles. The remaining unbound GA was quenched by incubation with 0.08 M glycine and 0.625 mg/mL NaBH_4_ for 30 min. The dissolution of the MnCO_3_ templates was performed by incubation with 0.5 M of EDTA at room temperature for 30 min. The obtained DOX-loaded HSA particles (DOX-HSA-MPs) were centrifuged, washed three times (10,000× *g* for 10 min) and finally suspended in sterile 0.9% NaCl. The control HSA-MPs were fabricated following the same procedures without doxorubicin incubation.

### 2.3. Particle Characterization

#### 2.3.1. Entrapment Efficiency of DOX

The amount of entrapped doxorubicin in DOX-HSA-MPs was calculated as the difference between the total amount of applied doxorubicin (DOX_t_) and the doxorubicin amount determined in the supernatant after absorption and after each washing step (ΣDOX_f_). The entrapment efficiency (EE%) was calculated according to the following Equation (1):

EE% = (DOX_t_ – ΣDOX_f_) × 100%/DOX_t_ (1)

The absorbance was measured with a microplate reader (PowerWave 340, BioTek Instruments GmbH) at 480 nm. The calibration curve is given as Figure S2 (Supplementary Materials).

#### 2.3.2. Particles Size and Zeta-Potential

The size and zeta potential of the DOX-HSA-MPs and HSA-MPs resuspended in PBS were measured by dynamic light scattering using a Zetasizer Nano ZS (Malvern Instruments Ltd., Malvern, UK) at 25 °C. All measurements were performed in triplicate and the mean values were taken as the result.

#### 2.3.3. Scanning Electron Microscopy (SEM)

The DOX-HSA-MPs and HSA-MPs were observed by SEM (LV-Scanning Electron Microscope, JSM 5910 LV, Tokyo, Japan). The samples were prepared by applying a drop of the particle suspension on a glass slide and then dried overnight at room temperature. Afterward, the samples were sputtered and coated with gold. SEM images were conducted at an operation voltage of 15 kV.

#### 2.3.4. Atomic Force Microscopy (AFM)

The morphology of HSA-MPs and DOX-HSA-MPs were investigated using AFM (Nano Wizard^®^4 Bruker, Berlin, Germany). The samples were diluted in water and spread on a clean coverslip. The drop was completely dried after incubation at 30 °C for 1 h and thereafter the cover slide was stored in dry conditions (air) for 4 days at 24 °C. The images of the particles were then taken (dry state). For the wet stage images the clean cover slip was covered with poly-l-ornithine, rinsed with water and dried by nitrogen flow. The samples were dropped on the coverslip and incubated for 30 min. Afterwards the samples were thoroughly washed and imaged in water. The JPK Data Processing software (version 6.1, Bruker Nano GmbH, Berlin, Germany) was used to analyze the obtained images.

#### 2.3.5. Confocal Laser Scanning Microscopy (CLSM)

HSA-MPs and DOX-HSA-MPs in suspension as well as after incubation with A 459 or BEAS-2B cells were investigated with a confocal laser scanning microscope (LSM 510 Meta, Carl Zeiss Micro Imaging GmbH, Jena, Germany) equipped with a 100× oil immersion objective (numerical aperture 1.3).

Images of autofluorescence of HSA-MPs and intrinsic DOX-fluorescence in DOX-HSA-MPs were taken using excitation wavelength 488 nm (Ar laser) and a long pass filter 505 nm. Cells additionally stained with Lysotracker^®^ Deep Red were imaged using excitation wavelength 633 nm (HeNe laser) and emission long pass filter 650 nm. For intracellular co-localization of DOX-HSA-MPs and Lysotracker^®^ Deep Red the particle fluorescence was detected using a band pass filter 530–600 nm.

#### 2.3.6. Release of DOX from DOX-HSA-MPs

For in vitro drug release, DOX-HSA-MPs were resuspended in two different media at a volume concentration of 3%. (i) PBS pH 7.4 and (ii) PBS pH 7.4 containing 0.2 mg/mL Pronase^®^ were placed into 2 mL microcentrifuge tube and incubated under shaking at 37 °C. At predetermined time intervals, the samples were centrifuged (10,000× *g*, 10 min) and the supernatant was collected for DOX quantification using a microplate reader (PowerWave 340, BioTek Instruments GmbH) at 480 nm. The release experiments were performed in triplicate, and the results were presented as mean ± SD. In parallel, the particles size was measured (Zetasizer Nano ZS).

### 2.4. Interaction of the Carriers with Cells

#### 2.4.1. Cell Culture

The cell line A549 is often used as an in vitro model of a lung cancer. Since albumin is endocytosed by alveolar epithelial cells via gp-60 albumin receptor [13,27,28,29,30,31] the A549 cell line is a useful model to study the cellular uptake of albumin particles. A549 cell line was cultured in RPMI 1640 medium supplemented with 10% FBS and 1% Penicillin-Streptomycin. Human bronchial epithelial cells (BEAS-2B) cell line was used as model for cells without albumin receptors. It was cultured in Bronchial Epithelial Basal Medium (BEBM^TM^) supplemented with 1% FBS. The cells were incubated at 37 °C in a humidified atmosphere of 5% CO_2_ incubator (Thermo Scientific, Waltham, MA, USA).

#### 2.4.2. Cytotoxicity Assay

In vitro colorimetric determination of cytotoxicity was assessed using Cell Counting Kit 8 (CCK-8). This assay kit measures the metabolic activity of dehydrogenases within the viable cells. The cells were seeded into 96-well culture plate at a concentration of 1000 cells/100 µL medium in each well and pre-incubated for 24 h to allow them to adhere. After this period, HSA-MPs and DOX-HSA-MPs were added to the cells at concentrations of 1000; 5000 and 10,000 particles/cell and incubated further for 24, 48 and 72 h at 37 °C, 5% CO_2_. Following these incubation times 10 µL of CCK-8 solution was added to each well and the plates were placed in the incubator for 1 h. Then the metabolic activity of the cells was assessed measuring the optical density at 450 nm using a microplate reader (PowerWave 340, BioTek Instruments GmbH). Percentage of metabolic activity was calculated by the mean absorbance of the samples at each incubation time point. The mean absorbance of the control cells at 24 h was set to 100% and used as a reference value. Afterwards, statistical evaluation of the data was performed using an analysis of variance (two-way ANOVA). Tukey’s multiple comparisons were used to compare the significance of differences between each sample and the control. A value of *p* < 0.05 was considered statistically significant.

#### 2.4.3. Cellular Uptake and Intracellular Localization of HSA-MPs and DOX-HSA-MPs

To investigate the uptake of particles, A549 cells were plated at a density 1×10^5^ cells/well in 24-well cell culture plates and incubated in a 5% CO_2_ humidified incubator at 37 °C for 24 h. The HSA-MPs and DOX-HSA-MPs particle suspensions (1000 and 5000 particles/cell) were then added and incubated for further 24 h. Following the incubation time, the cells were washed and fixed with 4% paraformaldehyde in PBS, washed again with PBS and incubated with a 0.25% trypsin solution for 5 min to harvest. After trypsinization, 1mL PBS was added per well and the total cell suspension was transferred for later reading. The percentage of cells with an uptake of particles was determined by flow cytometry (BD FACS Canto II, BD Biosciences, NJ) using the autofluorescence of the glutaraldehyde cross-linked protein for the HSA-MPs and the intrinsic fluorescence of DOX for the DOX-HSA-MPs, which was detected in the Allophycocyanin (APC)-channel (excitation 633 nm/emission > 660 nm). The uptake was additionally confirmed by Confocal Laser Scanning Microscopy (CLSM).

For intracellular localization of the HSA-MPs and DOX-HSA-MPs, the cells were additionally stained with 50 nM Lysotracker^®^ Deep Red 30 min before the end of incubation time. Then the cells were washed with PBS, fixed with 4% paraformaldehyde and observed by CLSM.

## 3. Results and Discussion

### 3.1. Fabrication and Characterization of DOX HSA-MPs

The fabrication process of DOX-HSA-MPs using MnCO_3_ as a template is shown in Figure 1. DOX is absorbed within the porous structure of the MnCO_3_-HSA-MPs mainly due to electrostatic and hydrophobic interactions. DOX can bind strongly to HSA via hydrophilic, hydrophobic contacts and hydrogen bonds [18,32]. Additionally, during the crosslinking with glutaraldehyde covalent cross-bounds are formed between amino groups of neighboring albumin molecules but also between albumin and adsorbed DOX which also contains an amino group. Finally, the MnCO_3_ template is dissolved with EDTA and the final DOX-HSA-MPs are obtained.

The dynamic light scattering measurement of MPs in PBS demonstrated an average size of HSA-MPs and DOX-HSA-MPs in the range between 700 to 800 nm (769 ± 39 and 793 ± 40 nm, respectively). No statistically significant differences of the hydrodynamic diameter of both particle types were found (Figure S1). In addition, the results demonstrated a low polydispersity index, indicating narrow size distribution of the particle population, which is in agreement with previously published data on HSA-MPs fabricated via the CCD-technique with MnCO3 as the inorganic template [24,25,26].

The zeta potential values of HSA-MPs and DOX-HSA-MPs measured in PBS were not significantly different (−13.4 ± 0.8 mV for both particle types). This result implies that the positively charged DOX was not presented on the surface but rather immobilized inside the porous HSA-MPs.

**Figure 2:**
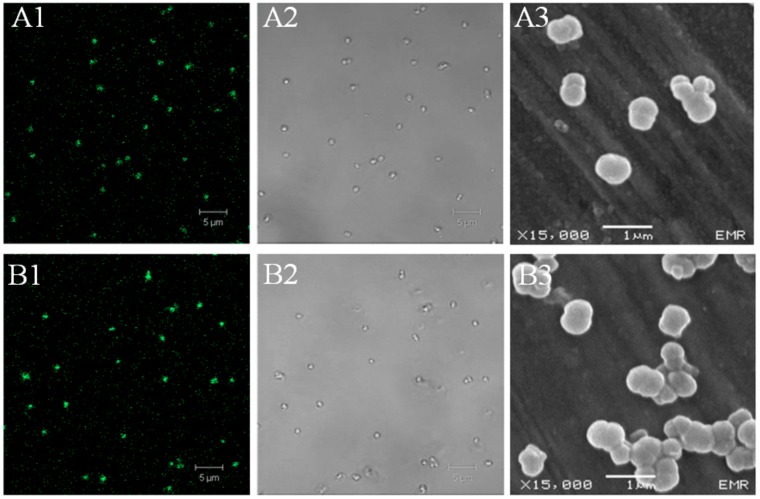
Microscopic images of HSA-MPs (**A1**–**3**) and DOX-HSA-MPs (**B1**–**3**). (**A1**,**A2**) Confocal Laser Scanning Microscopy (CLSM) images of HSA-MPs and (B1, B2) CLSM images of DOX-HSA-MPs in fluorescence mode (excitation wavelength 488 nm, long pass emission filter 515 nm) and transmission mode, respectively; (**A3**,**B3**), SEM images of HSA-MPs and DOX-HSA-MPs, respectively.

HSA-MPs (Figure 2A1) are visible under the CLSM due to their autofluorescence induced by glutaraldehyde [33]. DOX-HSA-MPs (Figure 2B1) show stronger fluorescence because of the intrinsic fluorescence of the incorporated DOX. The images in transmission mode show almost no particle aggregation confirming the low polydispersity index from the dynamic light scattering measurements of the size for both types of particles (Figure 2A2,B2). Scanning electron microscopy (SEM) also confirm the size of the particles in the submicron range (Figure 2A3,B3). Additionally, it is clearly seen that DOX has no influence on the particle shape and both, the HSA-MPs and DOX-HSA-MPs, have the typical peanut-like shape observed earlier for other preparations of protein particles using the CCD-technique with MnCO_3_ as a template.

**Figure 3:**
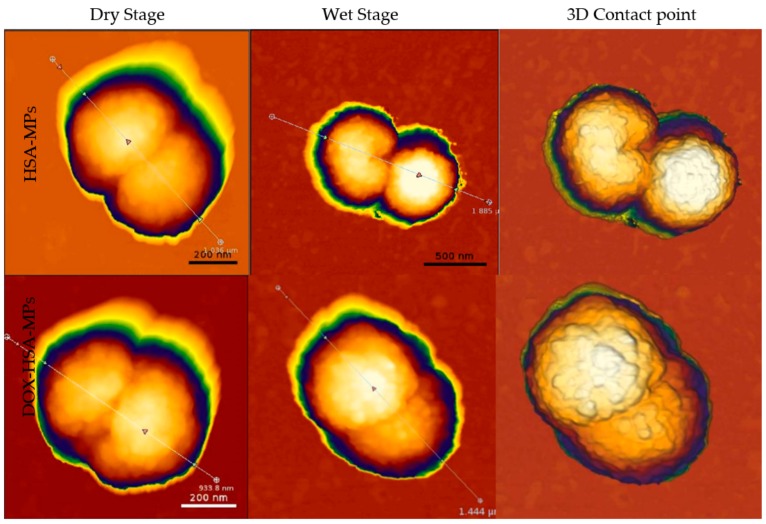
Atomic force microscopy images of HSA MPs and DOX-HSA-MPs particles in the dry as well as in the wet state (different scales) and as 3D contact point measurement.

The particles were further investigated by atomic force microscopy (AFM). Figure 3 shows images of AFM measurements illustrating the topography and size of particles in both dry and wet state. These measurements provide highly accurate determination of the particle dimensions and deliver additional information about the topography of their surface. The high-resolution AFM topography images also show the peanut-like shape of particles with submicron size and rough structures on the surface. The particles size was further determined under two different conditions. (i) In the dry state, HSA-MPs and DOX-HSA-MPs were in the same size range: length 690±50 nm and a height 480±40 nm. (ii) In the wet state, the size range of HSA MPs as well as of DOX-HSA-MPs was 950±60 nm (length) and 615±20 nm (height). The size increase in the wet state is due to the swelling of MPs and their sponge like behavior.

### 3.2. Entrapment Efficiency and Release of Doxorubicin from DOX-HSA-MPs

A high drug entrapment in drug carriers does not only reduce the necessary clinical dose, but also decreases the toxic side effects and improves the therapeutic effect [32,34]. In order to achieve possibly the highest concentration of DOX in the particles, doxorubicin hydrochloride was dissolved in 50% (*v*/*v*) DMSO in water and added to concentrated particle suspension (30% *v*/*v*). The entrapment efficiency for doxorubicin under these conditions was 25 ± 1.5% which corresponds to 7.25 fg DOX per particle.

**Figure 4:**
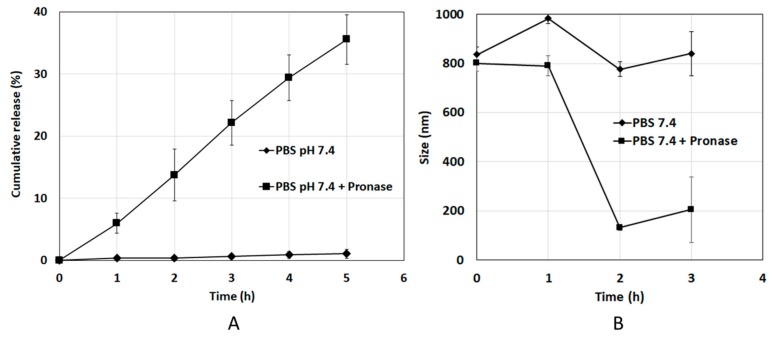
(**A**) Cumulative release profiles of DOX from DOX-HSA-MPs and (**B**) size change of DOX-HSA-MPs with time in the absence and presence of Pronase^®^ in PBS pH 7.4 at 37 °C. The values are displayed as mean and SD (*N* = 3).

DOX is bound to HSA and is not released if the DOX-HSA-MPs are resuspended in PBS (Figure 4A). Therefore, we investigated the release of DOX from the particles in an enzymatic environment to mimic the conditions encountered by the particles upon accumulation in the lysosomes. For this purpose, a proteolytic degradation of DOX-HSA-MPs particles by the mixture of proteases Pronase^®^ (0.2 mg/mL) was performed. As shown in Figure 4A, there is no release of DOX under enzyme-free conditions. However, the DOX-HSA-MPs released up to 38% of the entrapped DOX within 5 h in presence of Pronase^®^. Simultaneously the particle size during the release experiment was also assessed (Figure 4B). It can be seen there is no significant change of the size of DOX-HSA-MPs under enzyme-free conditions. However, already after 2 h incubation with Pronase^®^ the average size of the DOX-HSA-MPs was reduced from approximately 880 nm to 100 nm, suggesting a continuous digestion of the HSA in the particles, which consequently leads to the release of DOX.

### 3.3. Interaction of HSA-MPs and DOX-HSA-MPs with tumor cells

#### 3.3.1. Cytotoxicity to Tumor Cells

**Figure 5:**
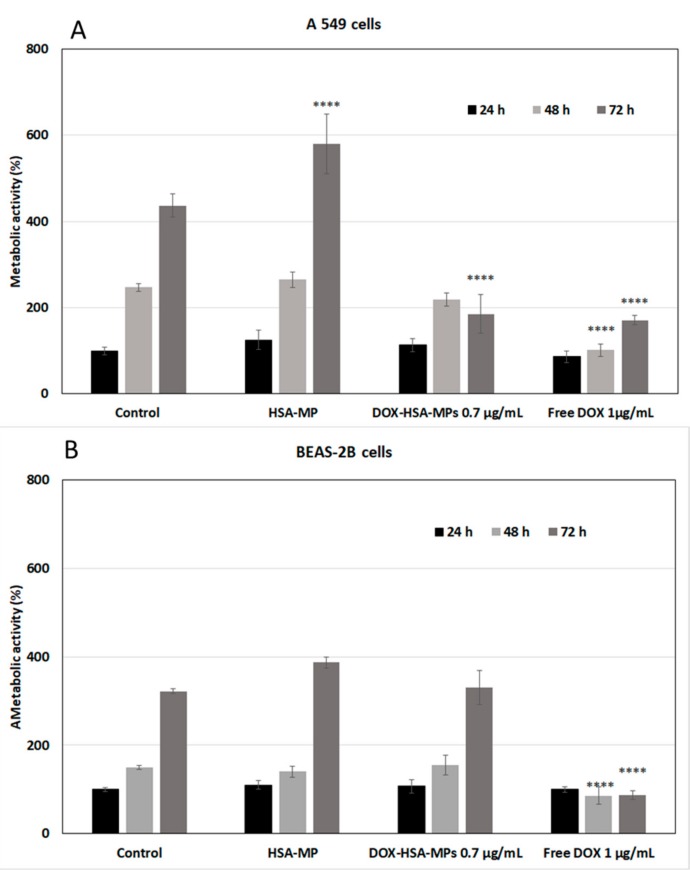
Metabolic activity of A549 lung tumor cells (**A**) and BEAS-2B bronchial epithelial cells (**B**) treated with HSA-MPs, DOX-HSA-MPs (10,000 particles per cell, corresponding to 0.725 µg/mL DOX) and free DOX (1 µg/mL) for 24, 48 and 72 h compared to untreated cells (control) using the Cell Counting Kit-8 (CCK-8) assay. The data are represented as two way ANOVA, Tukey’s multiple comparisons. The error bar represents mean ± SD; **** *p* < 0.001. The metabolic activity of the control after 24 h was taken as 100%.

A549 cell line has been widely used as a model system for the development of drug therapies against lung cancer. The cytotoxicity of HSA-MPs and DOX-HSA-MPs was examined by determining their effects on the mitochondrial metabolic activity of the cells after 24, 48 and 72 h exposure. At the lower particle concentrations (1000 and 5000 particles per cell), no significant differences between the metabolic activity of the cells cultured with both types of particles and that of the untreated cells were found (Figure S3, Supporting Information). However, after 72 h exposure to the highest particle concentration of 10,000 particles per cell the cell metabolic activity in the group treated with DOX-HSA-MPs was significantly decreased (Figure 5A). Remarkably, the effect on the A549 cells caused by the DOX-HSA-MPs containing a total DOX dose of 0.725 µg/mL (72 pg/cell, related to the seeded cell number) was almost the same as that was achieved by 1 µg/mL (100 pg/cell, related to the seeded cell number) free DOX added to the cell culture medium. However, free DOX reduced the metabolic activity significantly already after 48 h treatment. The faster effect of free DOX was expectable in the context, that the time-consuming uptake of the particles and their digestion by the cells were necessary to release the drug.

In parallel, the DOX-HSA-MPs were also cultured with the non-cancerous cell line BEAS-2B derived from normal human bronchial epithelium. Here, also 1000, 5000 and 10,000 particles were added per seeded cell. The CCK-8 assay resulted in no significant differences between the cells cultured with DOX-HSA-MPs and untreated cells (Figure 5B, Figure S4).

Our particles consist of albumin, which is known to have an impact on the metabolic activity, cell proliferation and survival of cells by an interaction with such as co-factors, hormones, growth factors, lipids, amino acids, metal ions, reactive oxygen and nitrogen species [35].

In the samples where the A549 cells were cultured for 72 h with HSA-MPs the metabolic activity was increased in comparison with that of the untreated cells. This could be understood as an extra nutrition that the cells receive after the uptake and digestion of the particles. In case of the DOX-HSA-MPs, the digestion leads to the release of DOX which interferes with the DNA [36] and decreases the cells metabolic activity.

Our results are in concordance with a previous report about Abraxane (Abr), which is an albumin nanoparticle-bound Paclitaxel (PTX), a chemotherapy agent. It has been highlighted that Abr exhibited a greater effect for the treatment of non-small-cell lung cancer compared to free PTX. The authors found that albumin downregulates the protein glucosamine 6-phosphate *N*-acetyltransferase 1 (GNA1) causing proliferative delay and cell adhesion defects in A549 cells and leading to the superior drug effect of Abr [37].

#### 3.3.2. Cellular Uptake

**Figure 6:**
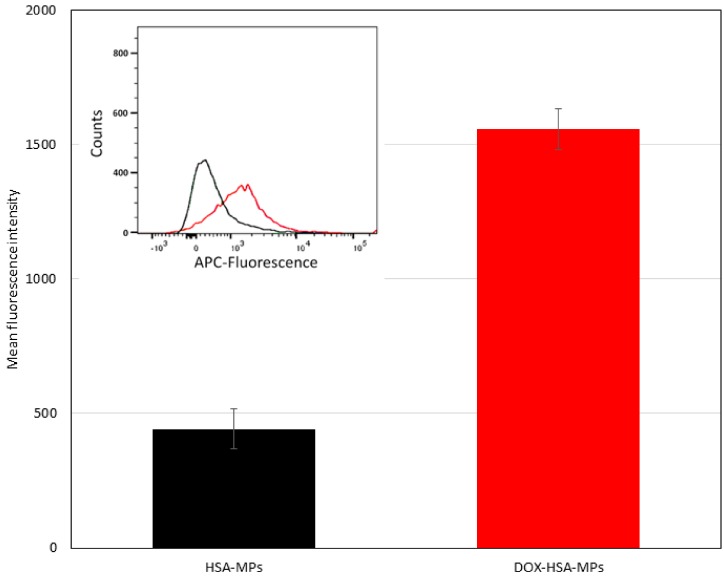
Mean fluorescent intensity (MFI) of HSA-MPs and DOX-HSA-MPs in the Allophycocyanin (APC) channel. Insert: Flow cytometry histograms showing the APC-fluorescence intensity distribution of the two types of particles (black line—HSA-MPs; red line—DOX-HSA-MPs in the.

For the quantitative determination of the cellular uptake of HSA-MPs and DOX-HSA-MPs we applied flow cytometry. As already observed by CLSM, both types of particles can be visualized by their intrinsic fluorescence if excited at 488 nm. For flow cytometry, we applied the APC channel in order to avoid influences by intrinsic fluorescence of the cells, which possibly can occur after their fixation with formaldehyde and is more intense if excited with wavelengths in the blue range. To clearly demonstrate the cellular uptake, we first determined the mean fluorescence intensity and distribution of the fluorescence intensity inside the particle populations (Figure 6). It can be seen that the DOX-HSA-MPs have a stronger fluorescence with a maximum over 1500 a.u. The HSA-MPs have a very weak fluorescence with a maximum around 500 a.u. which makes the quantitative determination of their uptake by the cells difficult. This was taken into account in the determination of the percentage of cells with uptake of particles.

**Figure 7:**
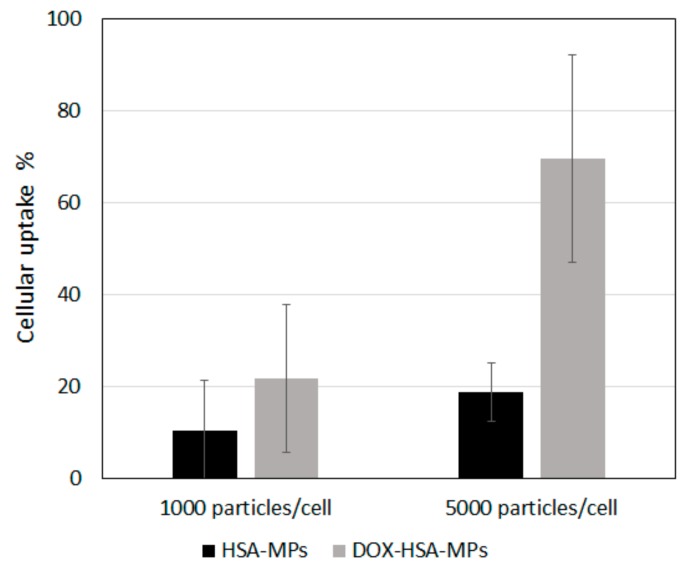
Cellular uptake of HSA-MPs and DOX-HSA-MPs (1000 and 5000 particles/cell) by A549 cells after culturing for 24 h.

In Figure 7 the results of the cellular uptake experiments, which were conducted by incubating A549 cells with the particles in two different concentrations (1000 and 5000 particles/cell) for 24 h, are summarized. At the lower particle concentration, the percentage of cells with uptake of HSA-MPs and DOX-HSA-MPs was 10.3% and 21.7%, respectively. At the higher particle concentration, the percentage of cells with uptake of DOX-HSA-MPs was increased more than 3-fold (69.5%), whereas the cells with uptake of HSA-MPs was only doubled (21%). However, due to the significantly lower mean fluorescence intensity of the HSA-MPs the uncertainty of the obtained values is high and it is not possible to claim that the DOX-HSA-MPs are preferably internalized by the A549 cells.

**Figure 8:**
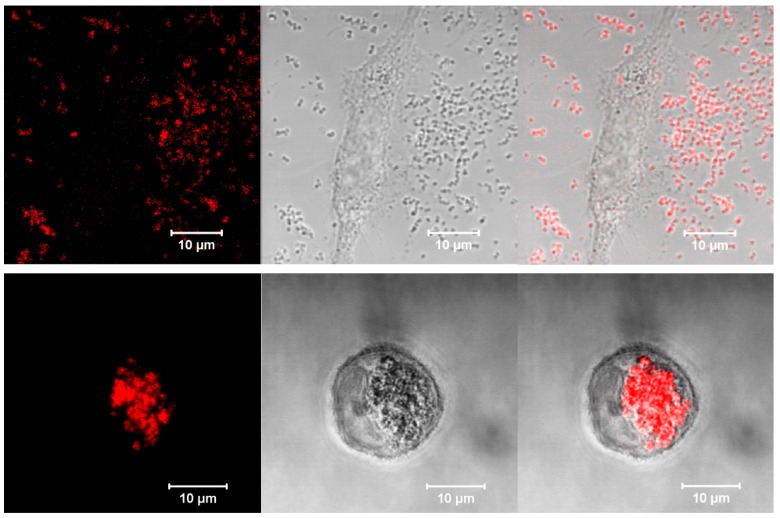
Representative CLSM images in fluorescence, transmission and overlay modes of a Beas-B2 cell (upper panel) and an A549 cell (lower panel) after 24 h culturing with DOX-HSA-MPs (5000 particles per cell). Fluorescence mode was accessed with excitation of DOX at 488 nm and long pass emission filter 530 nm.

To determine the location of particles in the cell compartments, A549 and BEAS-2B cells were incubated with DOX-HSA-MPs (5000 particles/cell) for 24 h and studies by CLSM. Figure 8 shows representative images of both samples. Obviously, BEAS-2B cells do not interact with the particles. A large number of DOX-HSA-MPs is distributed around the cell and close to the cell surface but inside the cells there are almost no particles (Figure 8, upper panel).

The images of the A549 cells incubated with DOX-HSA-MPs demonstrate a strong uptake (Figure 8, lower panel) with almost no particles in the space outside the cells, which is in accordance to the results obtained by flow cytometry, presented above.

#### 3.3.3. Intracellular localization

**Figure 9:**
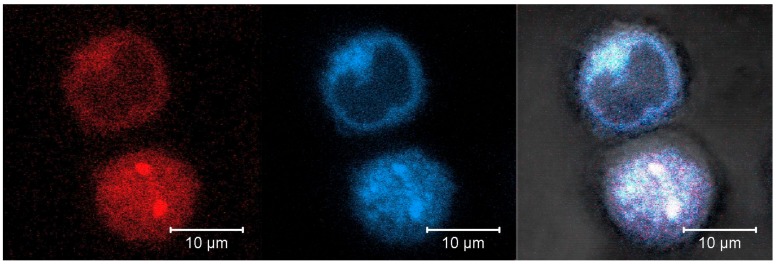
Representative CLSM images in fluorescence, transmission and overlay modes of A549 cells after 24 h culturing with DOX-HSA-MPs (5000 particles/cell) and Lysotracker^®^ Deep Red staining. Fluorescence modes was accessed with excitation at 488 nm and band pass emission filter 530/600 nm for DOX (red color) and excitation at 633 nm and long pass emission filter 650 nm for Lysotracker^®^ Deep Red (blue color).

As already shown above (Section 3.2) DOX is released from the particles only after their digestion by proteolytic enzymes. Therefore, the intracellular fate of the DOX-HSA-MPs is crucial for their efficacy as a drug carrier. A possible pathway of proteolytic degradation of the particles is their incorporation into the lysosomal compartment of the cells, where more than 60 different types of enzymes [38] are included. Therefore, we performed experiments with additional labelling of the A549 cells with Lysotracker^®^ Deep Red, a lysosomal marker. After incubation for 24 h with 5000 particles per cell the samples were studied by CLSM. A representative image is shown in Figure 9.

It can be seen that two DOX-HSA-MPs are internalized by one of the imaged cells. The overlapping of the red (DOX) and blue (Lysotracker^®^ Deep Red) color confirms co-localization of both fluorophores and therefore the co-localization of particles and lysosomes. This localization is important for the degradation of the particles by lysosomal enzymes and for the release of the drug from the degraded particles.

The possible pathway of the proteolytic degradation and intracellular localization of DOX-HSA-MPs (graphical abstract) is as follows. The DOX-HSA-MPs are firstly taken up at cell surface via endocytosis. Then, lysosomes and endosomes fuse to form autolysosomes and the scavenged particles are digested by lysosomal enzymes (more than 60 different types of enzymes) [38]. After digestion DOX and other breakdown products are released into the cytoplasm compartment. DOX generates free radicals which interfere and damage the cellular membranes, DNA and proteins of the cells finally resulting in cell death.

## 4. Conclusions

We have successfully loaded DOX into protein particles via CCD technique. The DOX-HSA-MPs exhibited a submicron size with negative zeta potential. The DOX entrapped into protein particles around 25% which is likely due to hydrophilic, hydrophobic contacts and hydrogen bond between DOX and albumin in the MnCO_3_ template. Remarkably, the release of DOX from the DOX-HSA-MPs was suppressed under physiological conditions (PBS pH 7.4). However, in the presence of proteolytic enzymes DOX was released to roughly 40% from the particles. The DOX-HSA-MPs showed higher efficacy inhibiting the metabolic activity of A549 cells at lower dose than free DOX after treatment for 3 days, which correlated well with their localization in the lysosomal compartment. In addition, no uptake and toxic effects of DOX-HSA-MPs were found when the particles were cultured with the non-cancerous cell line BEAS-2B. This suggests that our carriers are a highly promising drug delivery system for an alternative chemotherapy treatment of cancer.

## Supplementary Materials

The following are available online at https://www.mdpi.com/1999-4923/12/3/224/s1, Figure S1: Size distribution of HSA-MP and DOX-HSA-MP shown by numbers and by intensity. No statistically significant differences of the hydrodynamic diameter of both particle types were found. In addition, the results demonstrated a low polydispersity index, indicating narrow size distribution of the particle population and no aggregation of particles, Figure S2: Calibration curve for Doxorubicin dissolved in 50% DMSO, Figure S3: The cytotoxicity of HSA-MPs and DOX-HSA-MPs was examined by determining their effects on the mitochondrial metabolic activity of the cells after 24, 48, and 72 h exposure. At the lower particle concentrations (1000 and 5000 particles per cell), no significant differences between the metabolic activity of the cells cultured with both types of particles t and that of the untreated cells were found, Figure S4: In parallel, the DOX-HSA-MPs were also cultured with the non-cancerous cell line BEAS-2B derived from normal human bronchial epithelium. Here, also 1000, 5000 and 10,000 particles were added per seeded cell. The CCK-8 assay resulted in no significant differences between the cells cultured with DOX-HSA-MPs and untreated cells.
