# Supplementary material for: Doxorubicin–Loaded Human Serum Albumin Submicron Particles: Preparation, Characterization and In Vitro Cellular Uptake
Source: Pharmaceutics. 2020 Mar 2;12(3):224. doi: 10.3390/pharmaceutics12030224 (PMC7150780; doi:10.3390/pharmaceutics12030224)
Supplement: Supplementary file 1 [file pharmaceutics-12-00224-s001.pdf]

pharmaceutics

MDPI

Supplementary Materials: Doxorubicin–Loaded Human Serum Albumin Submicron Particles: Preparation, Characterization and In Vitro Cellular Uptake

Saranya Chaiwaree, Ausanai Prapan, Nittiya Suwannasom, Tomás Laporte, Tanja Neumann, Axel Pruß, Radostina Georgieva and Hans Bäumler

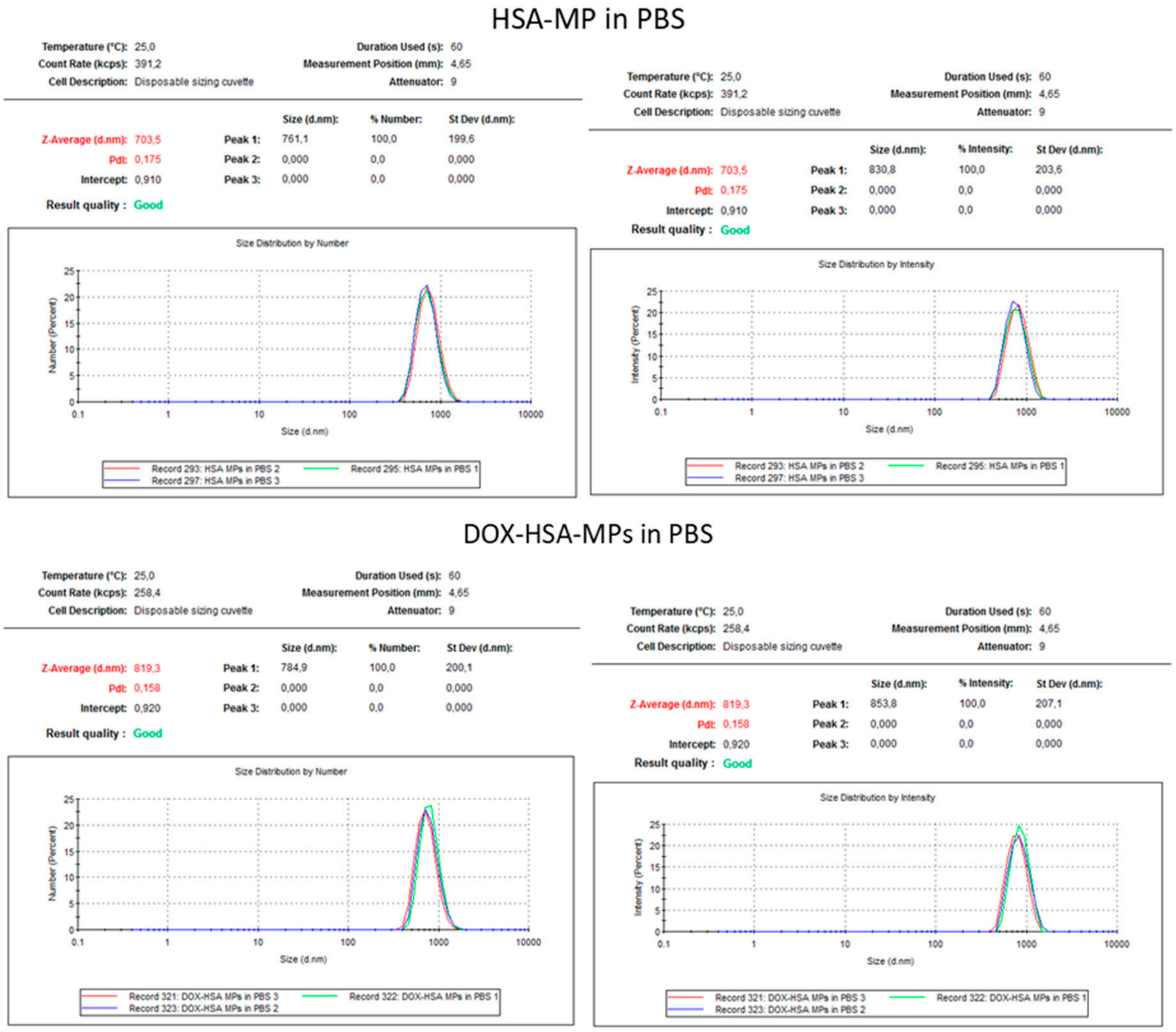

Figure S1. Size distribution of HSA-MP and DOX-HSA-MP shown by numbers and by intensity. No statistically significant differences of the hydrodynamic diameter of both particle types were found. In addition, the results demonstrated a low polydispersity index, indicating narrow size distribution of the particle population and no aggregation of particles.

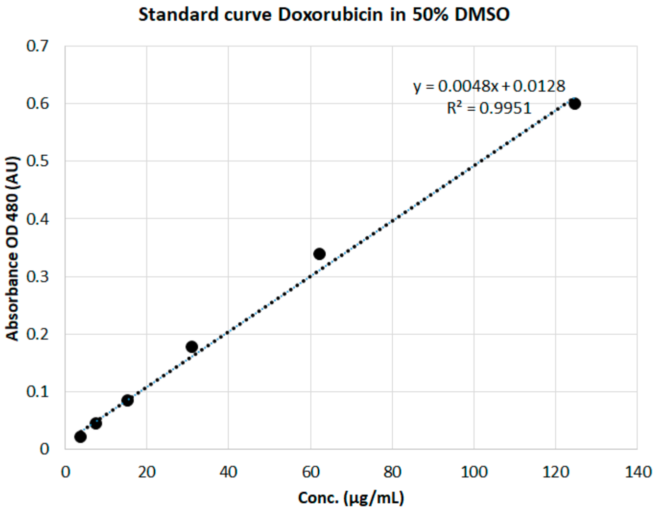

Figure S2. Calibration curve for Doxorubicin dissolved in 50% DMSO.

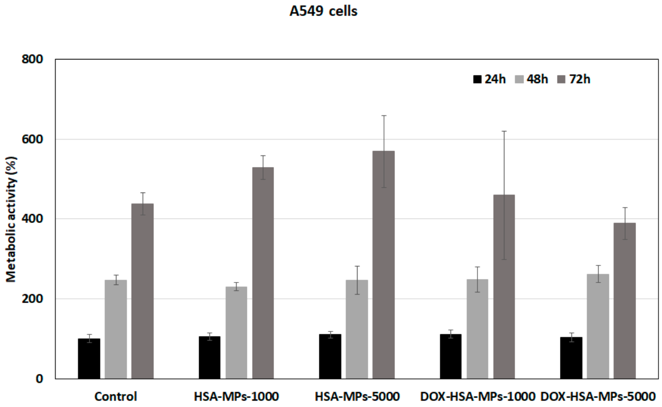

Figure S3. The cytotoxicity of HSA-MPs and DOX-HSA-MPs was examined by determining their effects on the mitochondrial metabolic activity of the cells after 24, 48, and 72 h exposure. At the lower particle concentrations (1000 and 5000 particles per cell), no significant differences between the metabolic activity of the cells cultured with both types of particles t and that of the untreated cells were found.

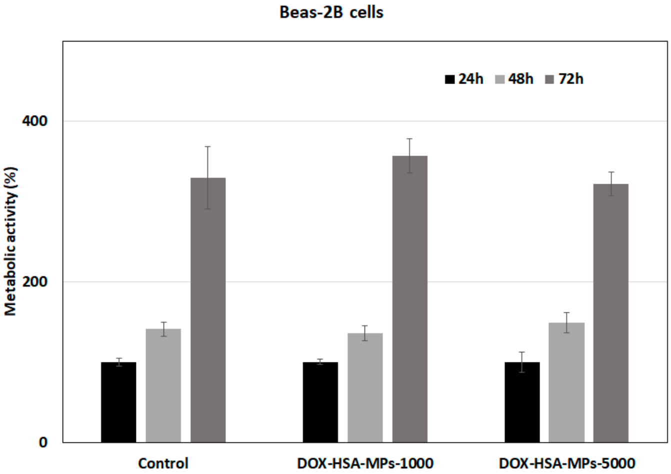

Figure S4. In parallel, the DOX-HSA-MPs were also cultured with the non-cancerous cell line BEAS-2B derived from normal human bronchial epithelium. Here, also 1000, 5000 and 10,000 particles were added per seeded cell. The CCK-8 assay resulted in no significant differences between the cells cultured with DOX-HSA-MPs and untreated cells.
